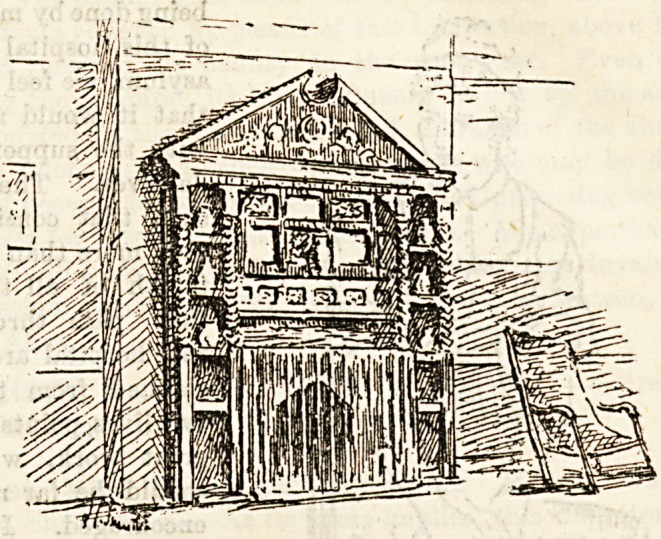# Asylum for Idiots

**Published:** 1891-01-03

**Authors:** 


### ASYLUM FOR IDIOTS.

**Earlswood Asylum for Idiots.**—This institution is a standing protest against the too great rush and hurry of the nineteenth century in regard to children, and an example of what may be achieved by applying with infinite patience a well thought out scheme of routine education to a dwarfed and stunted mind. Physically as well as mentally deficient, these poor children are trained in mind and body, every spark of progress eagerly welcomed and fostered, whatever natural capacity may lie hidden guided into useful channels till the helpless fingers, which could not lift a spoon to the mouth, slowly but surely turn to useful members. Sometimes it is not till after years of patient endeavour that the good seed shows its fruits, but the toil is rarely in vain, and the number of helpless children transformed to useful men and women is always increasing. The kind and courteous superintendent, Dr. Robert Jones, is untiring in his energetic efforts for the benefit of the helpless inmates of this home, who are not merely children, but those of larger growth, who, with whatever patient skill applied, can never fight their own battles in the world, but can have their miserable lot brightened by being trained to employ their fingers in useful occupation. It is supported mainly by voluntary subscriptions, and needs further assistance, which should be sent to Mr. James Dowring, Secretary, 36, King William Street, E.C.

## Figures and Tables

**Figure f1:**